# A Treatise on the Diseases of the Eye

**Published:** 1854-04

**Authors:** 


For sale by MILLER, ORTON & MULLIGAN.

---

ART. X.—*A Treatise on the Diseases of the Eye.* By W. LAWRENCE, F. R. S., Surgeon Extraordinary to the Queen, etc., etc. A new edition. Edited, with numerous additions and two hundred and forty-three illustrations, by ISAAC HAYS, M. D., Surgeon to Wills Hospital, etc., etc., etc. Philadelphia: Blanchard & Lea. 1854.

This is a large octavo of some 950 pages, devoted to this specialty. We can only speak of the general characteristics of the book, which strike us very favorably after the cursory examination given it.

It is, we believe, the fullest work upon the subject — the wood cuts are very numerous and highly illustrative — and it has evidently been the intention to make this new edition as perfect as the well-known ability of the American editor could render it.

But we do not like to speak at length of a book which we have not read, and it is hardly to be expected that even a reviewer's insatiate appetite should be able to digest the ten thousand little details of a work on ocular surgery, at a single sitting. But the work, though we ourselves have no personal acquaintance with it, is widely known in the profession as a very trustworthy authority, coming from a good source, and very complete and minute in its details.

The American editor has revised it (the author having declined that task) and has endeavored to bring it fully up to the latest improvements.

